# Nuclear Resonance Photon Scattering Studies of *N*_2_ Adsorbed on Grafoil and of *NaNO*_2_ Single Crystal

**DOI:** 10.6028/jres.105.022

**Published:** 2000-02-01

**Authors:** R. Moreh, Y. Finkelstein, D. Nemirovsky

**Affiliations:** Physics Department, Ben-Gurion University of the Negev, Beer-Sheva 84105 Israel; Nuclear Research Center, Negev, Beer-Sheva Israel; Physics Department, Ben-Gurion University of the Negev, Beer-Sheva 84105 Israel

**Keywords:** effective temperature, gas adsorption, lattice modes, (n,γ) reaction, normal modes, nuclear resonance photon scattering, zero-point energy

## Abstract

The nuclear resonance photon scattering (NRPS) from ^15^N_2_ adsorbed on graphite was investigated. The resonantly scattered intensities from the 6324 keV level of ^15^N with the photon beam parallel and perpendicular to the adsorbing grafoil planes was measured at 140 K and coverages below 0.7 monolayers (ML), where the ^15^N_2_ occur in the *vapor phase.* The data were used for deducing the out-of-plane tilt angle of adsorbed N_2_ relative to the graphite surface and the results were compared with grand canonical Monte Carlo (GCMC) calculations. Using the same method, a single crystal of NaNO_2_ was studied by measuring the scattering intensities with the nitrite planes aligned parallel and perpendicular to the photon beam. At 80 K, a huge anisotropy (R≈3.6) was observed, caused by the anisotropy in the zero-point motion of the internal modes of vibration of the NO_2_ ion. The variation of the scattering intensity from a powdered isotopic ^15^NaNO_2_ sample versus *T* in the range 12 K to 297 K was also measured and explained by accounting for the internal and external vibrational modes in NaNO_2_.

## 1. Introduction

The Doppler broadening of nuclear levels caused by the zero-point vibrations and thermal motion have been used for measuring the zero-point kinetic energies and linear momenta of atoms in solids and in adsorbed molecules on surfaces. This was done using nuclear resonance photon scattering (NRPS) from the 6324 keV level of ^15^N in N-containing molecules [1], and was applied for studying molecular orientations [2] in a variety of anisotropic systems [3–6]. We hereby report the results of two recent studies.

Physical adsorption of N_2_ monolayers on graphite (in the form of Grafoil) is probably the most studied system in relation to 2-dimensional (2D) physics; it was extensively studied using several techniques, such as n-diffraction, low-energy-electron diffraction, x-ray diffraction, adsorption isotherms and specific heats [7–11]. These studies yielded the in-plane orientational ordering and phase diagrams at temperatures below the 2D tricritical point (*T*_c_≈85 K), i.e., in the solid and liquid phases. One interesting feature, almost uncovered by the above experimental techniques, is the study of the *out-of-plane* tilt angle of the N_2_ molecular axis relative to the adsorbing graphite planes. At high temperatures, all diffraction techniques fail to yield any useful information concerning this topic. In this respect, the NRPS technique [1] is unique as it is the only technique which provides direct information on the tilt of N_2_ molecules relative to the graphite planes, not only in the fluid phase but also in the vapor phase.

**Fig. 1:**
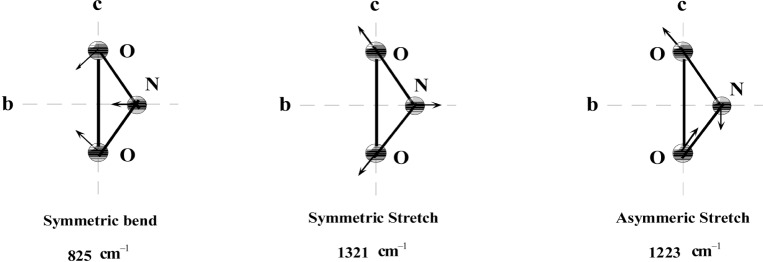
Internal normal modes of ${\mathrm{NO}}_{2}^{-}$ taken from Ref. 12]. Vectorial arrows represent atomic motions. Note that all modes are confined to the ${\mathrm{NO}}_{2}^{-}$ plane (*b,c*).

NaNO_2_ is a molecular solid; the nitrite ions ( ${\mathrm{NO}}_{2}^{-}$) in a single crystal are all parallel to each other; the NaNO_2_ has nine vibrational modes [12]: three internal modes (825 cm^–1^ < *v* <1321 cm^–1^) confined to the ${\mathrm{NO}}_{2}^{-}$ *ionic plane* (Fig. 1), and six external modes of the lattice (of Na^+^ against ${\mathrm{NO}}_{2}^{-}$) which occur in the 120 cm^–1^ to 220 cm^–1^ spectral region. The internal modes (which are all planar) are the main contributors to the zero-point motion, making the single crystal highly anisotropic. It is interesting to find out to what extent this anisotropy could be reproduced by experiment.

### 1.1 The NRPS Technique

**Fig. 2:**
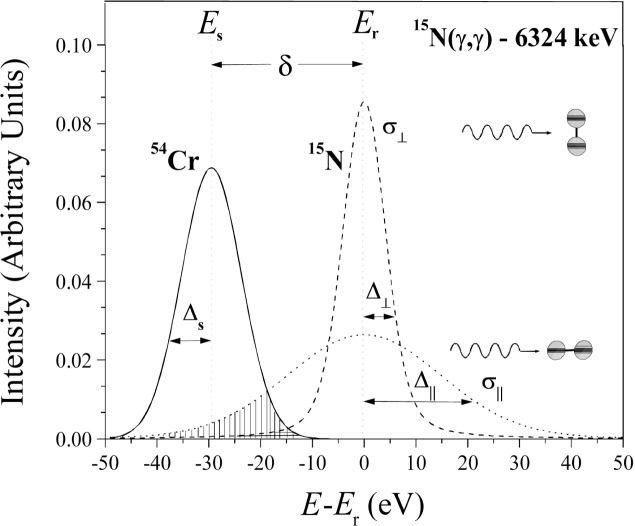
Calculated shape of the Doppler-broadened level at 6324 keV in ^15^N of peak energy *E*_r_ and of the Doppler-broadened incident line of the ^53^Cr(n, γ) reaction of peak energy *E*_s_ (after recoil correction), for *T*_s_ = 460 K. The nuclear level shape is depicted for ideal cases calculated at *T* = 0 K, with the molecular N_2_ symmetry axis positioned parallel and perpendicular to the photon beam. The corresponding Doppler widths *∆*_s_, *∆*_‖_ and *∆*_⊥_ of the three lines are indicated. The overlap integrals between the shape of the incident line and the nuclear level are shown as shaded areas and are related to the scattering cross sections *σ*_‖_ and *σ*_⊥_.

The basic idea of the NRPS method relies on monitoring the Doppler broadening of the nuclear level in ^15^N caused not only by the thermal motion but also by the internal *zero-point* vibrational motion of the N-atom. This technique uses a photon beam generated by the Cr(n,γ) reaction with neutrons obtained from a nuclear reactor. It so happens that the γ-line of ^54^Cr overlaps by chance [1] (within (*δ* = 29.5 eV) the 6324 keV nuclear level of ^15^N. The overlapping process is such that the resonance scattering cross section is proportional to the Doppler broadening of the nuclear level, *∆*_r_ = *E*(2*kT*_r_/*M*_r_*c*^2^)^1/2^, where *E*, the excitation energy, *M*_r_, the nuclear mass, *T*_r_, the effective temperature of the scattering atom, *k*, the Boltzmann constant, and *c* the velocity of light. It may be noted that *T*_r_ expresses the *total kinetic* energy of the scattering atom, including the part associated with its internal zero-point vibrational motion. This situation is schematically illustrated in Fig. 2 for the parallel and perpendicular orientations of the N_2_ molecular axis with respect to the γ-beam direction. The diatomic N_2_ molecule is highly anisotropic; the total kinetic energy of the N-atom is maximum along the N_2_ molecular axis (containing the internal vibrational motion) and minimum in the perpendicular direction. Hence the Doppler broadening of the ^15^N nuclear level should have a maximum, *∆*_‖_, along the N_2_ symmetry axis and a minimum, *∆*_⊥_, along the perpendicular direction. The corresponding scattering cross sections *σ*_‖_ and *σ*_⊥_ are proportional to the overlap integrals (shown as the shaded areas in Fig. 2) and fulfill the relation *σ*_‖_ ≫ *σ*_⊥_. Here, we utilize this dependence of the scattering cross section *σ*_r_ on the orientation of N_2_ with respect to the photon beam, for measuring the out-of-plane tilt angle of the N_2_ molecular axis with respect to the adsorbing graphite planes. Similarly, the anisotropy in the scattered intensities from the NaNO_2_ single crystal, reflects the anisotropy of the ${\mathrm{NO}}_{2}^{-}$ internal modes; all of which are confined to the nitrite ionic plane. Thus, the total kinetic energy of the N-atom has a maximum along the ${\mathrm{NO}}_{2}^{-}$ plane and a minimum along the normal to the plane.

## 2. Experimental Details

### 2.1 The γ-Source

The γ-source was generated from the (n, γ) reaction on three chromium disks (each 8 cm diameter and 1.5 cm thick) placed along a tangential beam tube near the core of the IRR-2 reactor. The photon beam was collimated and neutron filtered through 40 cm of borated paraffin before hitting the scatterer. The scattered photons were detected using two hyperpure Germanium (HPGe) detectors, with efficiencies of 35 % and 30 %, set 15 cm from the sample at scattering angles of 120°. The detectors were shielded against low-energy scattered photons and background neutrons using 9 mm lead and 1 cm borated plastic. Other details concerning the experimental system are found elsewhere [13].

### 2.2 The N_2_-Grafoil Sample

**Fig. 3:**
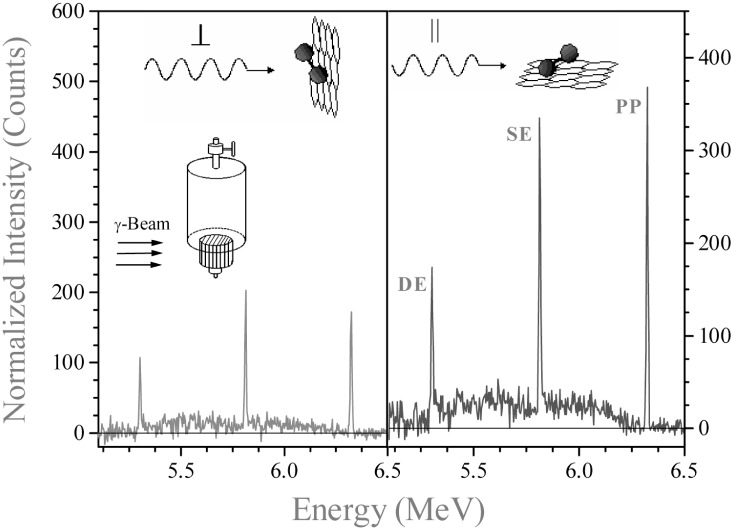
Scattered radiation spectra (background subtracted) from the N_2_+Grafoil sample at *T* = 20 K, with the γ-beam parallel (‖) and perpendicular (⊥) to the Grafoil planes, at a submonolayer coverage of 0.60 ML. The anisotropy ratio of the scattered intensities is *R* = *I*_‖_/*I*_⊥_ = 1.97. The photo peak, single-escape and double-escape peaks are labelled PP, SE, and DE. The inset shows the sample container where the path of the γ-beam is indicated. The upper gas reservoir is not hit by the photon beam.

The Grafoil cell consists of two thin-walled pure aluminum cylindrical compartments. The small one (40 mm i.d., 40 mm high, contains 40.5 g Grafoil consisting of 86 rectangular parallel sheets) is positioned in the path of the photon beam (see inset to Fig. 3), while the large one, serving as a gas reservoir, was outside the beam region. This design has the following advantages over the stainless steel cell of a previous work [2]: (i) the scattered background is reduced enormously. (ii) the scattered signal arises primarily from the adsorbed N_2_, while the contribution of the free non-adsorbed gas is very small. (iii) The Grafoil (purchased from Deutsch Carbon) is of a better quality in the sense that the product *f·ϕ* (where *f* = 0.30 is the randomly oriented fraction of the crystallite surfaces and *ϕ* = 30° the FWHM angle of the mosaic spread) is now smaller by ≈ 30 % compared to that of Ref. [2]. The cell was mounted inside a variable temperature cryostat (10 K to 300 K) which could be rotated around an axis coinciding with that of the Grafoil cylindrical cell from one position where the photon beam is parallel to the Grafoil planes of the sample to a perpendicular position. Isotopically enriched N_2_ (99 % ^15^N) was used.

### 2.3 The NaNO_2_ Samples

Two samples were used: a 1.03 g of *isotopic powdered* Na^15^NO_2_ sample (99 % ^15^N), placed in a thin walled, pure aluminum cylinder; it was used for measuring the scattered intensity versus *T.* The second, contained a ≈7 g *natural* NaNO_2_ single crystal (0.36 % ^15^N) having only ≈ 6 mg ^15^N, was employed for measuring the scattered intensity ratios *R* = *I*_‖_/*I*_⊥_ with the photon beam parallel and perpendicular to the ${\mathrm{NO}}_{2}^{-}$ planes of the single crystal whose orientation was determined using x-ray diffraction. Because of the very small amount of ^15^N (≈6 mg) present in the sample, the scattered background had to be reduced by fitting the outer and inner radiation shields of the cryostat by four 0.02 mm thick aluminum coated Mylar windows. The NaNO_2_ crystal was covered with thin Al foil to facilitate thermal conduction at low *T.* Because of some technical difficulties, we did not measure the scattering from the single crystal below 80 K. The temperature was monitored using two thermocouples set at two extreme points of the samples.

## 3. Theoretical Remarks

As discussed in Sec. 1.1, the resonance scattering cross section is proportional to the Doppler broadening of the nuclear level, which in turn depends on the scatterer effective temperature *T*_r._ It follows, that in order to predict the scattering cross sections, it was necessary to evaluate *T*_r_ of the N-atom for all scatterers in question. A detailed procedure for calculating *T*_r_ is given in Refs. [1] and [2]. We hereby give the method for evaluating the scattering cross section.

### 3.1 Resonance Scattering Cross Section

As illustrated schematically in Fig. 2, the resonance photon scattering process is caused by a Doppler broadened γ-line, represented by a Gaussian, *F*(*E*), overlapping a Doppler broadened nuclear level at 6324 keV in ^15^N, represented by a ψ-function ψ(*x,t*). The scattering cross section, for an infinitely thin ^15^N sample, is given by the overlap integral (shown as the shaded area in Fig. 2). This is expressed as [14]:

$$\sigma_{r}=\sigma_{0}\int_{0}^{\infty }F\left(E\right)\psi \left(x,t\right)dE=\sigma_{0}\psi_{0}\left(x_{0},t_{0}\right)$$ (1)

where *σ*_0_ = 2πλ^2^ *gΓ*_0_/*Γ* is the peak cross section of a nonbroadened nuclear level whose total natural width is *Γ* and ground state width *Γ*_0_. The Gaussian function *F*(*E*) is given by:

$$F\left(E\right)=\left(1/\Delta_{s}\pi^{1/2}\right)\exp\left[-{\left(E-E_{r}+\delta \right)}^{2}/\Delta_{s}^{2}\right]$$ (2)

where *∆*_s_ is the Doppler width of the incident line of the γ-source and is defined by:

$$\Delta_{s}=E_{s}{\left(2kT_{s}/M_{s}c^{2}\right)}^{1/2}.$$ (3)

*E*_s_ = *E*_r_−*δ* is the peak energy of the γ-line emitted by the ^53^Cr(n,γ) reaction; it is separated by an energy *δ* from the resonance energy *E*_r_ of the nuclear level (after recoil correction).

In this resonance scattering process, free recoil of the emitting nucleus is assumed; the recoil energy is: $E_{R}=E_{r}^{2}/2Mc^{2}=\;1.43\text{ keV}$ and is far larger than the lattice energies of the sample. *M*_s_ is the mass of the emitting isotope of the γ-source; *T*_s_ is the effective temperature of Cr defined by Lamb [15]. The function ψ(*x,t*) is a convolution between a Breit-Wigner resonance form and a Gaussian distribution of energies and is given by:

$$\psi \left(x,t\right)=\left(\frac{1}{2\sqrt{\pi }t}\right)\int_{-\infty }^{\infty }\frac{\exp\left[-{\left(x-z\right)}^{2}/4t\right]}{1+z^{2}}dz$$ (4)

$$\text{with}\;x=\frac{2\left|E-E_{r}\right|}{\Gamma },\;\;t={\left(\Delta_{r}/\Gamma \right)}^{2},\;\;\;\;\Delta_{r}=E_{r}{\left(2kT_{r}/M_{r}c^{2}\right)}^{1/2}$$ (5)

where, *E*_r_, *M*_r_ and *∆*_r_ are related to the scatterer and are defined in a similar manner to that of the γ-source. In Eq. (1), the overlap integral was expressed as another ψ function where [14]:

$$x_{0}=2\left|E_{r}-E_{s}\right|/\Gamma =2\delta /\Gamma \;\;\;\;\;\text{and}\;\;\;\;t_{0}=\left(\Delta_{s}^{2}+\Delta_{r}^{2}\right)/\Gamma .$$ (6)

Thus the scattering cross section depends strongly on the effective temperatures *T*_s_ and *T*_r_ through the corresponding Doppler widths. The nuclear parameters for the calculations were taken from Table I of Ref. [16]. The Doppler width of the incident 6324 keV line was taken to be 8.3 eV, corresponding to the actual temperature, *T ≈* 460 K, of the γ-source during reactor operation.

## 4. Results and Discussion

### 4.1 The N_2_+Grafoil Sample

**Fig. 4:**
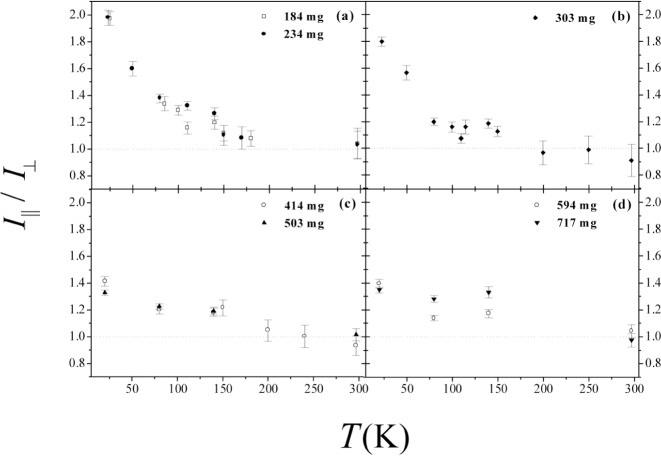
Measured ratios, *R* = *I*_‖_/*I*_⊥_, of the scattered intensities from the N_2_+Grafoil sample vs *T*. The initial gas amounts are indicated in each case: an amount of 303 mg of N_2_ corresponds to a complete commensurate monolayer (ML) on graphite. Other coverages correspond to submonolayer or to coverages higher than 1 ML. Dotted lines (*R* = 1) represent randomly oriented N_2_.

**Fig. 5:**
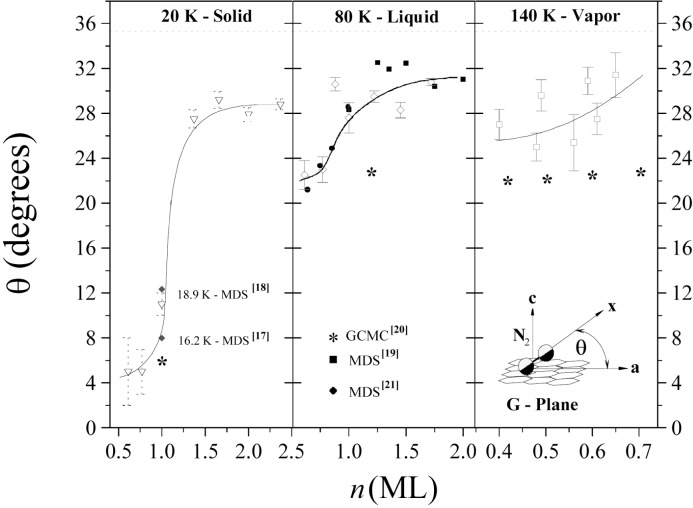
Deduced tilt angles, *θ*, of the N_2_ molecular axis with respect to the graphite planes as a function of molecular coverage for 20 K, 80 K, and 140 K (corresponding to the solid, liquid and vapor phases). Solid lines were passed through our measured data points (shown as open symbols with error bars) to lead the eye. The n-diffraction results at 10.5 K are labeled by full triangles. All other solid symbols (diamonds, circles, squares and stars) refer to theoretical calculations, where references are indicated. The inset at the bottom right corner defines the out-of-plane tilt angle *θ* of N_2_ relative to the graphite planes.

The spectra of the resonantly scattered intensities from the 6324 keV level of the ^15^N-containing samples have the same features but differ in intensity and in the signal/noise ratio. In Fig. 3 we show typical scattering spectra after background subtraction from the N_2_+Grafoil sample, at 20 K, using a 150 cm^3^ HPGe detector, for the two perpendicular geometries of the cell. The scattering intensities reveal a high anisotropy, *R* = 1.97, which means that the adsorbed N_2_ molecules lay nearly flat on the graphite surface. Figure 4 summarizes the measured values of *R* vs *T* for the different initial gas amounts in the cell. A value *R* = 1.0 means that the N_2_ molecules are randomly oriented relative to the planes of the graphite foils. Figure 4 shows that *R* decreases significantly with increasing *T* and also with the amount of gas for coverages above 1 ML. Thus *R* decreases to *R ≈* 1.30 at 80 K and ≈ 1.20 at 140 K. This trend continues to *R* = 1.0 at *T* ≥ 180 K, where the N_2_ librational amplitude becomes very large. In the vapor phase, the N_2_ occurring in the Grafoil region consists of an adsorbed part and a free nonadsorbed part. Above 80 K, the adsorbed part was determined by measuring the scattered intensities from the Grafoil compartment as a function of *T*. The data were then corrected to account for the fact that the scattered intensity from a constant amount of N_2_ decreases with *T* [1,2]. We thus found that at 140 K the adsorbed gas fraction within the irradiated compartment is between 70 % and 85 %, depending on the initial gas amount; the remaining amount occurs as a free nonoriented gas. Accounting for these fractions, the out-of-plane tilt angle was deduced from the measured *R* vs *T* and molecular coverage using the procedure of Ref. [2]. The results are depicted in Fig. 5, which shows the out-of-plane tilt angle versus molecular coverage for the solid (20 K), liquid (80 K) and vapor (140 K) phases. An outstanding result which emerge from Fig. 5 is the pronounced forward tilt of the N_2_ molecule on the graphite surface at 140 K, where the gas is in the *vapor* phase and the N_2_ is believed to stick loosely to the graphite surface. In general, good agreement is obtained between the NRPS values at 20 K and those obtained by n-diffraction experiments at 10.5 K [7]. Furthermore, molecular dynamic simulations (MDS) [17–20] seem to reproduce our measured data nicely at 20 K and 80 K (Fig. 5). For coverages above *n* = 1 ML, some of the MDS tilt angles at ≈80 K are slightly higher than our measurements. It should be noted that the MDS values [19] have an estimated uncertainty of ±1.5° at around *θ* ≈ 30°. As for the vapor phase, no experimental data exist and the only available calculations, at 140 K, are those using the GCMC simulations [21] which yield lower values than our measured data (Fig. 5). This deviation is probably due to the fact that the GCMC calculations assume a geometrically flat surface which neglects the surface corrugations of graphite. This later factor is important and when included as was done in the MDS, increases the predicted tilt angle, bringing it in much closer agreement with the measured values. Furthermore, the MDS results at lower temperatures, 20 K and 80 K (Fig. 5) which agree nicely with the present data also yield larger tilt angles than those of the GCMC. It would be interesting to extend the MDS calculations to 140 K to find out whether the present NRPS tilt angles can also be reproduced.

### 4.2 The NaNO_2_ Samples

#### 4.2.1 Temperature Variation of the Scattered Intensities in NaNO_2_

**Fig. 6:**
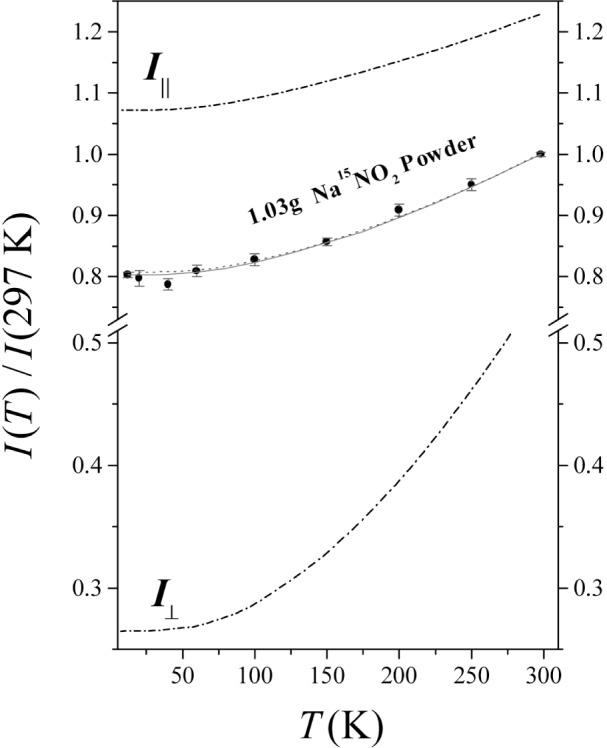
Temperature variation of the scattered intensities relative to 297 K from the 1.03 g powdered Na^15^NO_2_ sample. The solid line is a best fit calculated with a Debye temperature *Θ*_0_ = 320 K, while the dotted curve was calculated using the lattice frequencies [12]. The upper and lower curves are the calculated intensities *I*_‖_ and *I*_⊥_ relative to *I*(297 K) of the powdered sample.

The measured *T* dependence (relative to 297 K) of the scattered intensity *I*(*T*) from ^15^N in the isotopic powdered Na^15^NO_2_ sample is shown in Fig. 6; it drops monotonically with *T*, reaching a plateau below 50 K. This reveals a quantum effect caused by the zero-point motion of the N-atom in the crystal where the Doppler width of the 6324 keV level in the ^15^N scatterer reaches near its minimum value. The solid curve is calculated by assuming a Debye type behavior [22] for the external vibrational modes where a best fit to the data was obtained using a Debye temperature of *Θ*_0_ = 320 K. In another calculation (dotted curve) the external modes were taken from Ref. [12]. Both calculations reveal a good agreement with the data points. The other two lines of Fig. 6 (labeled *I*_‖_ and *I*_⊥_ show the calculated relative intensities with the photon beam set parallel and perpendicular to the nitrite plane of an assumed single crystal. The scattering intensities are defined as *I*_‖_ = (*I*_b_+*I*_c_)/2 and *I*_⊥_ = *I*_a_, where *I*_a_, *I*_b_, *I*_c_ are the scattering intensities along the *a*, *b*, and *c*-axes of the single crystal (where *b* and *c* are defined in Fig. 1 and a is along the normal to the (*b,c*) plane [12]). All curves in Fig. 6 are normalized to the scattered intensity from the powdered sample at 297 K denoted *I* (297 K), where *I* = (*I*_a_+*I*_b_+*I*_c_)/3. Note the huge decrease of *I*_⊥_ with *T* as compared with the relatively small drop of *I*_‖_. This again illustrates the effect of the zero-point motion which for the case of *I*_‖_ (representing the scattered intensity from the planar modes) is much larger than that of *I*_⊥_.

#### 4.2.2 Anisotropy Ratios in NaNO_2_ Single Crystal

**Fig. 7:**
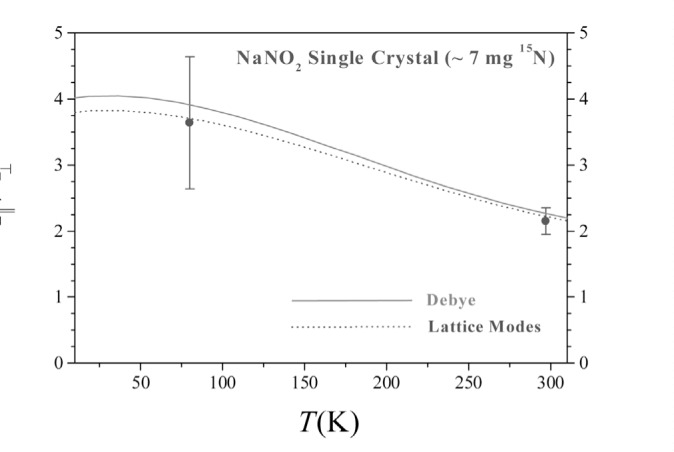
Measured scattered intensity ratios, *R* = *I*_‖_/*I*_⊥_ at 80 K and 297 K with the photon beam parallel and perpendicular to the *nitrite* planes of the single crystal. The solid and dotted curves correspond to calculations using the Debye and lattice mode models discussed in the text.

The measured scattered intensity ratios, *R* = *I*_‖_/*I*_⊥_, are given in Fig. 7 for 80 K and 297 K together with those calculated using a Debye temperature, *Θ*_0_ = 320 K (solid line). The dotted curve was calculated using the experimental lattice frequencies [12]. It may be seen that the above two calculations nicely reproduce the measured data points. Since we did not attempt to align the single crystal along either the *b* or *c* axis, we took the value of *I*_‖_ as *I*_‖_ = (*I_b_*+*I_c_*)/2. This procedure introduced an uncertainty of around 10 % in the calculated value which is far lower than the experimental error at 80 K. The relatively small error bar at 297 K is caused not only by the higher counting rate at 297 K but also because at 80 K, two cryostat radiation shields were used which increased the background at the detector.

#### 4.2.3 Comparison With Other Systems

It is interesting to compare the present results with other anisotropic systems such as C_24_Rb+^15^N_2_, in which the N_2_ molecules are adsorbed within the Rb planes of the graphite intercalation compound C_24_Rb. In this case, a high anisotropy ratio *R* ≈ 2.8 was measured [4] at 140 K; it is caused by the fact that the N_2_ molecules are adsorbed with their molecular axes nearly parallel to the graphite planes and because the C_24_Rb sample was prepared using highly oriented pyrolytic graphite (HOPG). In another example [3], ^15^NO_2_ gas was adsorbed on Grafoil where a large anisotropy was expected. However, only a small ratio *R ≈* 0.97 was measured between 297 K to 12 K. The reason could be traced down to the formation of dimers, N_2_O_4_, that are adsorbed with their N = N molecular axis perpendicular to the graphite planes which drastically reduced the anisotropy [3]. In yet another system, a *nitrate* Na^15^NO_3_ single crystal was used [23] and the measured ratio was ≈ 1.43 at 295 K, to be compared with *R* = 2.15 measured here for NaNO_2_. The reduced anisotropy is caused by the fact that one of the six internal modes of the planar ${\mathrm{NO}}_{3}^{-}$ ion is vibrating along the normal to the nitrate plane which increased the scattered intensity *I*_⊥_. This is in contrast to the *nitrite* case where all the *internal* modes are planar, with no component along the perpendicular direction.

## 5. Conclusions

We used the NRPS technique to study the molecular orientations in the N_2_+Grafoil system and in a single crystal of sodium nitrite. This was done by monitoring the zero-point motion of the N-atom, occurring in these two molecular forms. We have shown that the NRPS method is a powerful tool for measuring the properties of adsorbed molecules on graphite and for testing MDS and MC calculations of adsorbed gases. It may also be used for checking the phonon spectra in solids and the lattice modes in molecular crystals measured by infrared and Raman methods.

## References

[1] Moreh R, Shahal O, Volterra V (1976). Effect of molecular binding on the resonance scattering of photons from the 6.234 MeV in ^15^N. Nuc Phys.

[2] Moreh R, Shahal O (1986). Zero point energies and out-of-plane orientations of N2 adsorbed on Grafoil. Surf Sci.

[3] Moreh R, Finkelstein Y, Shechter H (1996). NO_2_ adsorption on Grafoil between 297 and 12 K. Phys Rev B.

[4] Moreh R, Pinto H, Finkelstein Y, Volterra V, Birenbaum Y, Beguin F (1995). Oriented N_2_ molecules in C_24_Rb. Phys Rev B.

[5] Moreh R, Shahal O (1989). Orientation of N_2_O molecules adsorbed on grafoil using resonance γ-ray scattering:. Phys Rev B.

[6] Moreh R, Fogel M (1994). Anisotropic γ-ray resonance scattering from a zinc crystal and the uncertainty principle. Phys Rev B.

[7] Wang R, Wang SK, Taub H, Newton JC, Shechter H (1989). Multilayer structure of nitrogen adsorbed on graphite. Phys Rev B.

[8] Diehl RD, Fain SC (1983). Structure and orientational ordering of nitrogen molecules physisorbed on graphite. Surf Sci.

[9] Morishage K, Mowforth C, Thomas RK (1985). Orientational order in CO and N_2_ monolayers on graphite studied by X-ray diffraction. Surf Sci.

[10] Larher Y (1978). “Phase transitions” between dense monolayers of atoms and simple molecules on the cleavage face of graphite, with particular emphasis on the transition of nitrogen from a fluid to a registered monolayer. J Chem Phys.

[11] Chung TT, Dash JG (1977). N_2_ monolayers on graphite: specific heat and vapor pressure measurements—thermodynamics of size effects and steric factors. Surf Sci.

[12] Hartwig C, Wiener Evnear E, Porto SPS (1972). Analysis of the temperature-dependent phonon structure in sodium nitrite by Raman spectroscopy. Phys Rev B.

[13] Moreh R, Shahal O, Jacob I (1974). Study of the temperature effect of resonantly scattered capture γ-rays. Nuc Phys.

[14] Arad B, Ben-David G, Pelah I, Schlezinger Y (1964). Studies of highly excited nuclear bound levels using neutron capture gamma rays. Phys Rev.

[15] Lamb WE (1939). Capture of neutrons by atoms in a crystal. Phys Rev.

[16] Finkelstein Y, Moreh R (1997). Effect of varying the temperature of γ-sources on the resonance scattering cross section. Nuc Inst and Meth in Phys Res B.

[17] Talbot J, Tildesely DJ (1986). A molecular dynamics simulation of the uniaxial phase of N2 adsorbed on graphite. Surf Sci.

[18] Migone AD, Kim HK, Chan MHW, Talbot J, Tildesley DJT, Steele WA (1983). Studies of the orientational ordering transition in nitrogen adsorbed on graphite. Phys Rev Lett.

[19] Vernov AV, Steele WA (1986). Computer simulation study of the multilayer adsorption fluid N_2_ on graphite. Langmuir.

[20] Talbot J, Tildesely DJ, Steele WA (1985). Molecular-dynamics simulation of fluid N_2_ adsorbed on a graphite surface, Faraday Discuss. Chem Soc.

[21] Bottani EJ, Bakaev VA (1994). The grand canonical ensemble Monte Carlo simulation of nitrogen on graphite. Langmuir.

[22] Moreh R, Levant D, Kunnoff E (1992). Effective and Debye temperatures of solid N_2_, N_2_O, N_2_O_2_ and N_2_O_4_. Phys Rev B.

[23] Shahal O, Moreh R (1978). Study of atomic velocities in molecules using nuclear resonance photon scattering. Phys Rev Lett.

